# High resolution image dataset by RGB and multispectral cameras on an unmanned aerial vehicle over a secondary tropical dry forest

**DOI:** 10.1016/j.dib.2023.109869

**Published:** 2023-11-27

**Authors:** Masuly Vega-Puga, Jaime Garatuza-Payán, Miguel A. Rivera-Diaz, Onésimo Galaz, Juan C. Alvarez-Yépiz, Enrico A. Yepéz

**Affiliations:** a Departamento de Ciencias del Agua y Medio Ambiente, Instituto Tecnológico de Sonora, 5 de Febrero 818 Sur, Cd. Obregón, Sonora 85000, México; b Laboratorio Nacional de Geoquímica y Mineralogía, Sede Regional Sur de Sonora, 5 de Febrero, 818 Sur, Cd. Obregón, Sonora 85000, México

**Keywords:** Land-use change, Remote sensing, Structure from motion, Orthomosaic

## Abstract

Unmanned Aerial Vehicles (UAV) are advantageous to assess vegetation and terrain changes at a high spatial resolution, advancing our understanding of shifts in ecosystem states and processes. The extension of land that we sought to observe belongs to the footprint of an Eddy Covariance System (around 7.5 ha), installed in a secondary ecological succession of the tropical dry forest (27.00598005 N, 108.77913821 W) within the Sierra de Álamos-Rio Cuchujaqui natural protected area, Sonora, Mexico. Aerial images were obtained with a system of multispectral and RGB cameras mounted on a UAV, which executed automated missions in two different seasons: 1) dry season (June 2023) and 2) first rain season (July 2023), totalling 1116 images. The UAV mission was flown at a height of 105 m and images included a side and front overlap of 70%. The final product was an image dataset per season. These data can contribute to the continuous monitoring strategy to understand ecosystem processes and conserve the Sierra de Álamos-Rio Cuchujaqui natural protected area, a critical natural protected area for national and international organizations. Likewise, image datasets are useful for the processes of developing regional models of phenology, structure, richness, distribution, and vegetation cover along secondary succession after the forest was changed to agriculture and livestock land.

Specifications Table

**Table:** 

Subject	Environmental Sciences, Computers in Earth sciences
Specific subject area	Photogrammetry
Data format	RGB images Raw Image size: > 1000 KB Image composite color: RGB (Red, Green, Blue) Byte - 8 bits integer Multispectral images Raw Image size: 4.2 MB Multispectral image: Blue, 450 nm ± 16 nm; Green, 560 nm ± 16 nm; Red, 650 nm ± 16 nm; Red Edge: 730 nm ± 16 nm; Near Infra, 840 nm ± 26 nm. Byte - 16 bits integer
Type of data	Image format: JPEG and TIFF Geotagged image format: EXIF format Image resolution: 1300 × 1600 pixels Image coordinate system: Lat/Long Image datum: WGS84
Data collection	Images were captured with a 1/2.9 CMOS sensor array, RGB, and monochrome sensors, mounted on a DJI Phantom 4 multispectral UAV. The UAV executed an automated mission programmed in the DJI GS Pro-application (Flight monitoring interface, iPad mini-4). Parameters flight: a) 105 m height, b) 70% side and front overlap, c) 4.80 ha covered area, d) - 90 gimbal pitch angle, and f) 18 km/hour speed. 588 images per season (dry and first rain), were obtained; 98 RGB images and 490 multispectral.
Data source location	Organization: Instituto Tecnológico de Sonora. Site: The site is located at the Reserva Monte Mojino, a private protected natural area managed by Naturaleza y Cultura Internacional (https://www.natureandculture.org) that relies within the Área de Protección de Flora y Fauna Sierra de Álamos–Río Cuchujaqui in the municipality of Álamos, SonoraCity, Town/region: Álamos, Sonora, Mexico [1]. Central point (eddy covariance tower): 27.00598005° N y −108.77913821° W
Data accessibility	Repository name: Mendeley Data Data identification number: DOI:10.17632/rrs7ctswft.2 Direct URL to data: https://data.mendeley.com/datasets/rrs7ctswft/2

## 1 Value of the Data

• The dataset of RGB and spectral images in both seasons (dry and first rain) provides the opportunity to characterize the tropical dry forest in a secondary stage of succession [2], using the photogrammetry products, such as, orthomosaics [3].
• The data could be useful for monitoring changes of the ecosystem, not only in terms of vegetation but also of terrain, through digital terrain and surface models obtained from processing the data set with photogrammetry, such as vegetation structure and phenology, mortality patters and terrain characteristics.
• The data set can be useful for organizations that aim to conserve or manage natural resources of the tropical dry forest such as the Comision Nacional de Areas Natrales Protegidas (CONANP) and Nature Culture International (https://www.natureandculture.org/mexico) whom actively carry conservation activities at the Area de Proteccion de Flora y Fauna Sierra de Alamos Rio Cuchujaqui in northwestern Mexico [1].
• The dataset can be used for the characterization the morphology of vegetation, detection of individual trees, quantification of aerial biomass, biodiversity assessments and nutrient reservoirs.
• Fine spatial resolution of vegetation and terrain structural features from advanced remote sensing products aid to a better comprehension of dynamic controls of carbon and water fluxes in long-term monitoring observatories that relay on eddy covariance flux measurements [4].
• The data set can be used as classroom material for environmental and computer science careers.

## 2 Data Description

The image datasets are organized in two folders in the repository [5], according to the seasons in which the monitoring missions of the dry tropical forest occurred (dry and first rain season). The Dry Season folder has two subfolders: a) Dry Season RGB, with 98 images in JPEG format, and b) Dry Season Multi, with 490 multispectral images in TIFF format. Also, the First rain season folder has two subfolders: a) Rain season RGB, with 98 images in JPEG format, and b) Rain season Multi, with 490 multispectral images in TIFF format.

## 3 Experimental Design, Materials and Methods

**Fig. 1:**
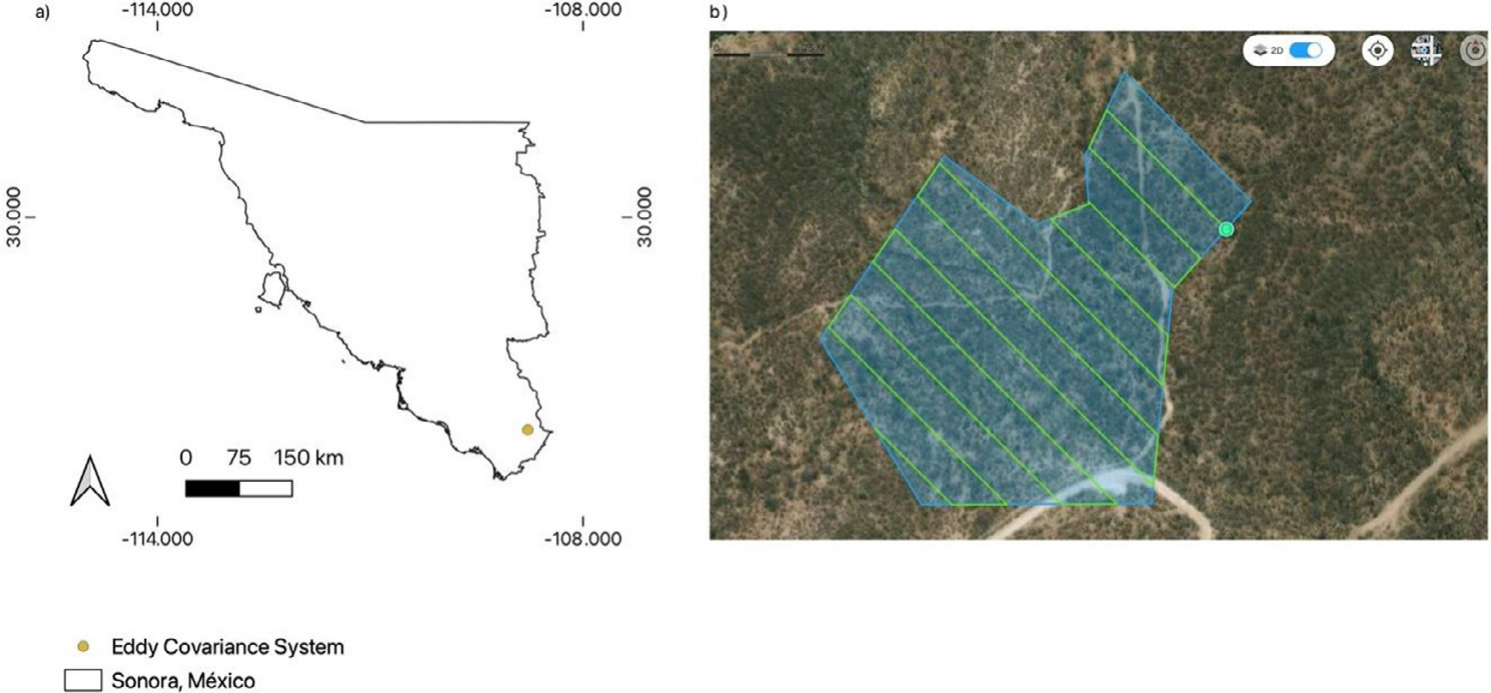
a) Location of Eddy Covariance site in southern Sonora, Mexico, and b) automated mission by DJI GS Pro-to fly the site.

The study site is a secondary tropical dry forest within the Sierra de Álamos-Rio Cuchujaqui natural protected area, Sonora, Mexico. The climate is warm and semiarid with a mean temperature of 24 °C and a mean annual precipitation of 712 [6]. The tree canopy is heterogeneous ranging heights between 9 and 15 m, with an important presence of legume species such as *Acacia cochliacantha* and *Lysiloma divaricatum,* and the genera *Bursera* [6]. This region presents two dominant seasons, a) dry season, from November to June, where is possible to see the terrain characteristics since the vegetation is deciduous, and b) a rainy season from July to October, which is principally driven by the North America Monsoon [7]. To obtain the image datasets of the dry (June 2023) and first rain (July 2023) season, a Phantom 4 Multispectral UAV (SZ DJI Technology Co, Shenzhen, Guangdong, China) equipped with a six-camera system (1/2.9 CMOS, RGB, and monochrome) was used. The UAV was programmed with its native application (DJI GS Pro) to execute an automated mission that flew over most of the area of influence of the eddy covariance tower installed in the secondary ecological succession of a tropical dry forest (coordinates: 27.005951 N, - 108.779084 W), as can be seen in the Fig. 1. The flight parameters were a) 105 m height, b) 70% side and front overlap, c) 98 waypoints, d) 4.80 covered area, e) −90 gimbal pitch angle, f) 18 km/hour speed, and g) 6 cm approximate spatial resolution. The established parameters were a function of the flux footprint from the eddy covariance tower, in order to acquire an adequate spatial resolution for landscape analysis, as well as the importance of meeting a high percentage of overlap to use the photogrammetric technique, Structure from Motion and Multi-View Stereo [8,9].

## Limitations

Not applicable.

## Ethics Statement

We have read the ethical requirements for publication of the article in Data in Brief.

This study does not involve human subjects, animal experiments and any data collected from social media platforms.

## CRediT authorship contribution statement

**Masuly Vega-Puga:** Conceptualization, Methodology, Software, Data curation, Writing – original draft. **Jaime Garatuza-Payán:** Supervision, Writing – review & editing. **Miguel A. Rivera-Diaz:** Methodology, Supervision. **Onésimo Galaz:** Methodology, Software. **Juan C. Alvarez-Yépiz:** Supervision, Writing – review & editing, Funding acquisition. **Enrico A. Yepéz:** Conceptualization, Writing – review & editing, Funding acquisition.

## Data Availability

RGB and Multispectral images dataset (Álamos, Sonora) (Original data) (Mendeley Data)
